# Outer Membrane Vesicles from the Probiotic *Escherichia coli* Nissle 1917 and the Commensal ECOR12 Enter Intestinal Epithelial Cells via Clathrin-Dependent Endocytosis and Elicit Differential Effects on DNA Damage

**DOI:** 10.1371/journal.pone.0160374

**Published:** 2016-08-03

**Authors:** María-Alexandra Cañas, Rosa Giménez, María-José Fábrega, Lorena Toloza, Laura Baldomà, Josefa Badia

**Affiliations:** 1 Secció de Bioquímica i Biología Molecular, Departament de Bioquímica i Fisiologia, Facultat de Farmàcia, Universitat de Barcelona, Barcelona, Spain; 2 Institut de Biomedicina de la Universitat de Barcelona, Barcelona, Spain; Laurentian, CANADA

## Abstract

Interactions between intestinal microbiota and the human host are complex. The gut mucosal surface is covered by a mucin layer that prevents bacteria from accessing the epithelial cells. Thus, the crosstalk between microbiota and the host mainly rely on secreted factors that can go through the mucus layer and reach the epithelium. In this context, vesicles released by commensal strains are seen as key players in signaling processes in the intestinal mucosa. Studies with Gram-negative pathogens showed that outer membrane vesicles (OMVs) are internalized into the host cell by endocytosis, but the entry mechanism for microbiota-derived vesicles is unknown. *Escherichia coli* strains are found as part of normal human gut microbiota. In this work, we elucidate the pathway that mediate internalization of OMVs from the probiotic *E*.*coli* Nissle 1917 (EcN) and the commensal ECOR12 strains in several human intestinal epithelial cell lines. Time course measurement of fluorescence and microscopy analysis performed with rhodamine B-R18-labeled OMVs in the presence of endocytosis inhibitors showed that OMVs from these strains enter epithelial cells via clathrin-mediated endocytosis. Vesicles use the same endocytosis pathway in polarized epithelial monolayers. Internalized OMVs are sorted to lysosomal compartments as shown by their colocalization with clathrin and specific markers of endosomes and lysosomes. OMVs from both strains did not affect cell viability, but reduce proliferation of HT-29 cells. Labeling of 8-oxo-dG adducts in DNA revealed that neither OMVs from EcN nor from ECOR12 promoted oxidative DNA damage. In contrast, flow cytometry analysis of phosphorylated γH2AX evidenced that OMVs from the probiotic EcN significantly produced more double strand breaks in DNA than ECOR12 OMVs. The EcN genotoxic effects have been attributed to the synthesis of colibactin. However, it is not known how colibactin is exported and delivered into host cells. Whether colibactin is secreted via OMVs is an open question that needs further study.

## Introduction

Intestinal microbiota has a great impact on human health. These microbial populations provide crucial benefits to the host, including metabolic activities, development of the host immune system, and prevention of gut colonization and infection by pathogens [1–3]. The intestinal epithelium is the first line of defence against pathogens and is also the surface where the host interacts with microbiota. It is protected by a mucus layer that prevents close contact between luminal bacteria and the epithelial surface [4]. Therefore, factors secreted by microbiota that can diffuse through the mucin layer, such as membrane vesicles, play a relevant role in intestinal communication. Extracellular vesicles are secreted by all bacteria. The best characterized are the outer membrane vesicles (OMVs) produced by Gram-negative bacteria. These vesicles are spherical, bilayered membrane structures that are released during normal bacterial growth and have sizes ranging from 20 to 250 nm. They act as a secretion pathway for a set of selected proteins and other active compounds in a protected environment. Bacterial vesicles have important biological functions in both bacterial survival and host interaction, allowing cell-to-cell communication without intimate intercellular contact. Depending on their cargo, they promote modulation or subversion of the host defence and immune responses [5,6]. A great number of studies performed with Gram-negative pathogens showed that OMVs are internalized in the host target cells, and contribute to virulence by delivering cytotoxic factors and mediators that interfere with the immune system [7–9]. In addition, OMVs isolated from several pathogenic *E*. *coli* strains and from the laboratory strain DH5α are genotoxic to human intestinal epithelial cells. Upon internalization, these bacterial vesicles can cause DNA lesions and affect cell proliferation and viability [10,11].

Uptake of pathogen-derived OMVs by epithelial host cells is mainly driven by endocytosis. This process involves invagination of the cell membrane, and takes place through different pathways depending on the composition and cargo of the vesicles to be internalized. There are two main endocytic pathways: clathrin-mediated endocytosis (CME), and the lipid raft-mediated pathway, which is cholesterol sensitive. These pathways produce endosomal compartments with different surfaces that allow the delivery of their cargo to various subcellular destinations [12]. CME involves a complex protein network including clathrin and dynamin as key components. Lipid rafts are dynamic membrane microdomains rich in cholesterol, sphingolipids and proteins such as caveolin and flotillin, which are associated with distinct clathrin-independent pathways. Vesicles from enterohemorrhagic *E*.*coli* enter host cells via CME [13], whereas vesicles from enterotoxigenic *E*. *coli*, *Porphyromonas gingivalis*, *Moraxella catharralis* or *Pseudomonas aeruginosa* are internalized through a lipid raft-mediated pathway in a clathrin-independent manner [14–17]. Clathrin-mediated endocytosis is the main pathway involved in the uptake of *Helicobacter pylori* OMVs, but lipid microdomains also contribute, although to a lesser extent [18].

Nowadays, microbiota vesicles are perceived as key players in the maturation of the immune system and in signaling processes at the intestinal mucosa [9,19]. However, studies in this field are still scarce, and to our knowledge there are no reports to date on the internalization pathway of vesicles produced by gut microbiota or probiotic strains.

*Escherichia coli* strains are commonly found as part of normal human gut microbiota. Some of these strains, such as *Escherichia coli* Nissle 1917 (EcN), provide benefits to the host, especially due to their ability to compete and inhibit gut colonization by pathogens. EcN is a good colonizer of the human gut and positively affects gastrointestinal homeostasis and microbiota balance. It is used as a probiotic in the treatment of intestinal disorders, especially inflammatory bowel diseases [20,21]. EcN upregulates the expression of both antimicrobial peptides and tight junction proteins, and promotes anti-inflammatory modulation of the immune response [22–25]. This probiotic strain is closely related to the uropathogenic *E*. *coli* strain CFT073, but lacks genes encoding particular virulence factors such as haemolysin and P-fimbrial adhesin. Both strains belong to the phylogenetic group B2, and although they cause different effects on the host (benefit or disease), their functional gene profiles are very similar [26,27]. We have recently reported the proteomic analysis of OMVs produced by the probiotic strain EcN. We identified 192 vesicular proteins with functions related to adhesion, immune modulation or bacterial survival in host niches [28]. Some of these proteins can target the vesicles to the host, and mediate their beneficial effects on intestinal function. We have also shown that EcN-derived OMVs modulate the expression of several cytokines and regulators of the intestinal barrier in human colonic explants, and in co-cultures of epithelial Caco-2 cells/peripheral blood mononuclear cells. Upon OMVs internalization, the activated epithelial cells elicit a response in the underlying immunocompetent cells [29].

In the present study, we elucidated the internalization pathway of EcN OMVs in several human intestinal epithelial cell lines, and examined whether these vesicles promote cytotoxic or genotoxic effects. We extended the analysis to other commensal *E*. *coli* strains without known probiotic traits, such as ECOR12 that belongs to phylogenetic group A, which is largely associated with non-pathogenic *E*. *coli* strains.

## Materials and Methods

### Bacterial strains and growth conditions

*Escherichia coli* Nissle 1917 (EcN) (serotype O6:K5:H1) was provided by Ardeypharm GmbH, Herdecke, Germany. ECOR12 is a commensal strain isolated from the stool of a healthy human [30]. Bacterial cells were routinely grown at 37°C in Luria–Bertani broth (LB) with constant rotation (150 rpm). Growth was monitored by measuring the optical density at 600 nm.

### Cell culture and growth conditions

The human colonic cells lines Caco-2 (ATCC HTB37) and HT-29 (ATCC HTB-38) were obtained from the American Type Culture Collection. The mucus-secreting HT29-MTX cell line was a gift from Thécla Lesuffleur, INSERM, Paris, France. Cells were cultured in DMEM High Glucose (Dulbecco’s Modified Eagle Medium) or DMEM Glutamax medium (Life Technologies), both supplemented with 10% (v/v) fetal bovine serum, 25 mM HEPES, 1% non-essential amino acids, penicillin (100 U/ml) and streptomycin (100 μg/ml) (Gibco BRL, MD, USA). Cultures were incubated at 37°C with 5% CO_2_. Cells were routinely subcultured once a week with trypsin-EDTA (0.25%, 0.53 mM) and seeded at a density of 2x10^5^ cells per 55 cm^2^ dishes.

### Isolation and labeling of OMVs

OMVs were isolated from culture supernatants as described previously [28]. Briefly, bacterial cells were culture overnight and pelleted by centrifugation at 10,000×*g* for 20 min at 4°C; the supernatants were filtered through a 0.22 μm-pore-size filter (Merck Millipore) to remove residual bacteria and concentrated using a Centricon^®^ Plus-70 filter device (Merck Millipore). Vesicles were obtained by centrifugation at 150,000×*g* for 1 h at 4°C in an Optima ^™^ L-90K ultracentrifuge (Beckman Coulter), washed and resuspended in an adequate volume of phosphate buffered saline (PBS) and stored at -20°C. Protein concentration was determined by the method of Lowry et al. [31].

Vesicles were fluorescently labeled with rhodamine isothiocyanate B-R18 (Molecular Probes). Fluorescence of this probe is quenched in bilayer membranes at high concentration but it is dequenched when probe is diluted after membrane fusion. OMVs purified as described previously were washed, resuspended in labeling buffer (50 mM Na_2_CO_3_, 100 mM NaCl, pH 9.2) and incubated with rhodamine isothiocyanate B-R18 (1 mg/ml) for 1 h at 25°C, followed by centrifugation at 100,000x*g* for 1 h at 4°C. Subsequently, to fully remove the unbound dye, samples were washed with PBS (0.2 M NaCl) followed by ultracentrifugation at 100,000 x*g* for 1 h at 4°C. After a final centrifugation step, B-R18-labeled OMVs were resuspended in PBS (0.2 M NaCl) containing a protease inhibitor cocktail tablet (Complete Protease Inhibitor Tablet, Roche) and stored at 4°C for up to 6 weeks.

### Internalization of OMVs

To monitor OMVs entry by epithelial cells, a total of 1x10^5^ HT-29 cells were seeded in 96-well plate (Corning Incorporated, Costar^®^) and grown to 80% confluence. Prior to the assay, cells were washed once with PBS. The medium was replaced with rhodamine B-R18- labeled OMVs (2 μg/well) suspended in DMEM without fetal bovine serum and red phenol but containing gentamicin. Cells were incubated at 37°C and fluorescence was measured over time in a Modulus^™^ Microplate fluorescence reader (Turner BioSystems) (Ex 570 nm; Em 595 nm). Fluorescence intensity was normalized by fluorescence detected at the indicated time points by labeled-OMVs in the absence of cells. Internalization assays with Caco-2 or HT29-MTX cell lines were performed with confluent monolayers fully polarized (17 days post-confluence). During growth and differentiation the medium was changed every 2 days.

To determine the mechanism involved in the endocytosis process, monolayers of HT-29, HT29-MTX or Caco-2 cells grown in 96-well black plates were pre-treated with the endocytosis inhibitors: dynasore 80 μM, chlorpromazine 15 μg/ml, filipin III 10 μg/ml or nystatin 10 μg/ml (all from Sigma-Aldrich) for 1 h at 37°C prior to the addition of labeled-OMVs. Control cells were not treated with the inhibitors. Rhodamine B-R18- labeled OMVs (2 μg protein/well) were applied to the apical side. Cells were incubated at 37°C and fluorescence was tracking over the time. Cell samples were also analyzed by microscopy as described below to ensure that no cell damage was caused by the inhibitors at the concentration used and to confirm the intracellular location of labeled-OMVs.

### Confocal fluorescence microscopy

HT-29 cells were grown in 8-well chamber slider (ibidi) until approximately 80% confluence and incubated with rhodamine B-R18-labeled OMVs (2 μg/well) at 37°C or 4°C for 15min, 30 min, 1 h and 3 h and then washed with PBS. Nuclei were labeled with DAPI and plasma membrane with fluorescent wheat germ agglutinin (WGA). For this purpose, cells were incubated for 25 min with Alexa-488 WGA (1 μg/ml, 4°C; Molecular Probes) followed by fixation for 30 min with 3% paraformaldehyde. After PBS washing, cells were analysed microscopically. To determine colocalization of OMVs with clathrin, cells were incubated with rhodamine B-R18-labeled OMVs for 15 min at 37°C and clathrin was stained using anti-clathrin mouse monoclonal antibody (clone 23,BD Biosciences) and Alexa Fluor 488-conjugated goat anti-mouse IgG (Molecular Probes). In control experiments, cells were incubated for 15 min with transferrin-Alexa Fluor 633 (TF-633) at 20 μg/ml (Molecular Probes). TF-633 was used as a positive control for clathrin-mediated endocytosis. To determine colocalization of OMVs with the endo-lysosomal compartments, cells were incubated with rhodamine B-R18-labeled OMVs (2 μg/well) for 30, 45 and 60 min. Cells were fixed with 3% paraformaldehyde, permeabilized with 0.05% saponin (Sigma Aldrich) and blocked using PBS containing 1% bovine serum albumin. Endosomes were detected with rabbit polyclonal antibody against EEA1 (Abcam^®^) and Alexa Fluor 488-conjugated goat anti-rabbit IgG (Molecular Probes). Lysosomes were detected using LysoTracker Green DND-26 at 300 nM (Molecular Probes). When indicated, internalized OMVs were labeled with anti-*E*. *coli* LPS antibodies (Abcam^®^) and Alexa Fluor 546-conjugated anti-mouse IgG (Molecular Probes).

Confocal microscopy was carried out using a Leica TCS SP5 laser scanning confocal spectral microscope, using the 63x oil immersion objective lens. Images were captured with a Nikon color camera (16 bit). Fluorescence was recorded at 405 nm (blue; DAPI), 488 nm (green; WGA and Alexa Fluor 488), 546 nm (red; rhodamine isothiocynate B-R18, Alexa Fluor 543 and MitoTracker Red FM) and 633 nm (far-red; TF-633, Alexa Fluor 633). Z-stack images were taken at 0.5 to 1.0-μm. Images were analyzed using Fiji image processing package. Colocalization was assessed by calculating the overlap coefficient (r) from quantitative data obtained from four confocal stacks using the JaCoP plugin. The value of this coefficient ranges from 0 to 1. Data were presented as mean ± standard error from four independent experiments. In all cases, the total number of cells analyzed was between 90 and 130.

### Cell viability and proliferation assays

The trypan blue exclusion test was used to study the ability of OMVs to affect the proliferation of HT-29 cells. HT-29 cells plated into 24-well plates were exposed to 5 μg/ml OMVs for up to 168 h. As a rule, once every two days, the cells were trypsinised, stained with 0.25% w/v trypan blue, and counted with a haemocytometer. The Mean Proliferative Index (MPI) at any given point was calculated as described elsewhere [10]. MPI = (number of cells treated with OMVs in each well / number of control cells in each well) x 100. To estimate the cell proliferation rate we calculated the population doubling time (PDT) values according to the equation: PDT = CT/ log (N1/N0)x 3.31; where CT is the culture time, N1 the cell number at the end of cultivation period and N0 the cell number at the beginning of the experiment [32].

Cell viability was assessed by the MTT (3-(4,5-Dimethylthiazol-2-yl)-2,5-diphen yl tetrazolium bromide) assay, based on the mitochondrial reduction of tetrazolium to formazan. Formazan formation is considered directly proportional to viable cell numbers. For this, 1x10^4^ HT-29 cells in 100 μl of media were plated into each well in a 96-well plate and incubated for 24 h at 37°C before the addition of OMVs (5 μg/ml) and further incubated for up to 168 h. At one or two day intervals, the cells were treated with 0.25% MTT (Sigma-Aldrich) in PBS and allowed to react for 2 h at 37°C. The medium was then removed and 1 ml of solubilization reagent (99% dimethyl sulfoxide) was added (Applichem, Ecogen). Cell viability was measured at 570 nm in a Modulus^™^ Microplate Photometer (Turner BioSystems). The results were expressed as percentage of cell survival relative to the control (untreated cells).

### Cell cycle analysis

After treating HT-29 cells with OMVs (5 μg/ml) for 24, 48 and 72 h, cells were collected by centrifugation (1000×g, 3 min) and washed once with cold PBS. The pellet was resuspended in 0.5 ml cold PBS and 4.5 ml cold 70% ethanol and left at −20°C until use. Prior to analysis, cells were centrifuged, washed once with cold PBS, resuspended in PBS containing RNase A (200 μg/ml) and incubated for 20 min at room temperature. The cells were then permeabilized with 0.1% Triton X-100, stained with propidium iodide (5 mg/mL final concentration) for 1 h and analysed by flow cytometry using an Epics XL flow cytometer (Coulter Corporation, Hialeah, Florida). The instrument was set up with the standard configuration: excitation of the sample was done using an standard 488nm air-cooled argon-ion laser at 15mW power. Forward scatter (FSC), side scatters (SSC) and red (620 nm) fluorescence for PI were acquired. Optical alignment was based on optimized signal from 10 nm fluorescent beads (Immunocheck, Epics Division). Time was used as a control of the stability of the instrument. Red fluorescence was projected on a 1024 monoparametrical histogram. Aggregates were excluded gating single cells by their area vs. peak fluorescence signal. DNA analysis (Ploidy analysis) on single fluorescence histograms was done using Multicycle software (Phoenix Flow Systems, San Diego, CA). A total of 10,000 events were analysed for each sample.

### Immunofluorescence microscopy of mutagenic 8-oxo-dG adducts

Mutagenic 8-oxo-7,8-dihydro-2´-deoxyguanosine (8-oxo-dG) adducts are among the lesions generated by hydroxyl radical attack on DNA. Formation of 8-oxo-dG adducts in cells exposed to OMVs was analyzed by immunofluorescence microscopy using mouse monoclonal anti-8OHdG antibodies (Abcam^®^). HT-29 cells grown in 8-well chamber slider (ibidi) were exposed to 5 μg/ml OMVs for up to 168 h (7 days). As a control of this kind of oxidative damage, HT-29 cells were treated with 300 μM H_2_O_2_ for 24 h. Fluorescence detection of 8-oxo-dG in cell nuclei was evaluated by following the primary antibody manufacturer´s instructions. Briefly, the cells were washed with PBS, fixed with 3% paraformaldehyde, permeabilized using 0.5% Triton X-100 in PBS for 30 min at room temperature, blocked with 0.1% Triton X-100 in 5% Bovine serum albumin for 1 h at room temperature and then incubated using anti 8OHdG (Abcam^®^) for 16 h at 4°C. The secondary antibody was an Alexa-Fluor 488 conjugated goat anti-mouse antibody (Life Technologies). Nuclei were labeled with DAPI.

### Immunodetection of phosphorylated histone γH2AX

γH2AX is required for checkpoint-mediated arrest of cell cycle progression and for efficient repair of DNA double strand breaks (DSBs). Recruitment of phosphorylated histone γH2Ax to sites of damaged DNA in HT-29 cells exposed to OMVs versus HT-29 control cells was evaluated by using immunofluorescence microscopy and flow cytometer. The cells were treated with 5 μg/ml OMVs for up to 168 h (7 days) or with 300 μM H_2_O_2_ for 24 h (as positive control of DNA damage). For immunofluorescence microscopy, the cells were fixed with paraformaldehyde and blocked with BSA prior to incubation with anti-gamma H2A.X antibodies (Abcam^®^) for 16 h at 4°C. Samples were washed and incubated with the Alexa-Fluor 546 conjugated goat anti-rabbit secondary antibody for 1h at 37°C. Nuclei were labeled with DAPI.

Quantification of γH2Ax was performed by flow cytometry. HT-29 cells, plated on 6-well tissue culture plates, were exposed to 5 μg/ml OMVs for 7 days and then washed with culture medium to removed unbound OMVs. The cells were trypsinized, fixed in ice-ethanol and stored at -20°C until analysis. After three washes with PBS, the fixed cells were treated with RNAse (200 μg/ml) and stained with propidium iodide (10 μg/ml) for 30 min at room temperature. Then, cells were permeabilized with 0.5% Triton X-100 in PBS for 30 min and blocked with 5% BSA for 2 h at room temperature. Samples were incubated with anti-gamma H2A.X followed by Alexa-Fluor 546 conjugated goat anti-rabbit antibodies as described above. The cells were washed and analysed using a Beckman Coulter Cytomics FC500 flow cytometer. Cell debris and dead cells were excluded from analysis by gating cells using FSC *vs* SSC double dot. A total of 10,000 events were analyzed for each gated sample. Statistical analysis data were presented as mean ± standard error from at least three independent experiments.

### Evaluation of DNA damage by alkaline single-cell gel electrophoresis (Comet assay)

This method was used to assess the formation of DSBs in cells exposed to OMVs. Glass slides were coated with 1.5% agarose and then dried at room temperature. HT-29 cells treated with 5 μg/ml OMVs for up to 168 h or with 300 μM H_2_O_2_ for 24 h (positive control) were trypsinized, washed twice in PBS and mixed with low melting point agarose (1:5). The agarose suspended cells were spread on the coated slides, covered with 22 × 22 mm coverslips and left at 4°C for approximately 20 min to allow the agarose settle down. Cells were then lysed at 4°C for 2 h in lysis buffer (2.5 M NaCl, 100 mM EDTA, 10 mM Tris, 10% DMSO, 1% v/v Triton X-100, pH 10). The slides were transferred to a BioRad horizontal electrophoresis tank containing electrophoresis buffer (10 M NaOH, 0.2 M EDTA) and left for 40 min to equilibrate before being electrophoresed for 45 min at 25 Volts. After three washes in neutralisation buffer (0.4M Tris pH 7.5) the cells were stained with ethidium bromide (20 μg/ml). The slides were examined using a Leica D1000 microscope with a 63X oil immersion objective.

### Statistical analyses

Statistical analysis was performed using SPSS (version 20.0, Chicago, IL, USA) software package. All assays were repeated at least three independent times in triplicate. The values for all measurements are presented as the mean ± standard error. Differences between more than two groups were assessed using one-way ANOVA followed by Tukey’s test. The *p* value less than 0.05 were considered statistically significant.

## Results

### OMVs from probiotic and commensal *E*. *coli* strains are internalized by intestinal epithelial cells

**Fig 1:**
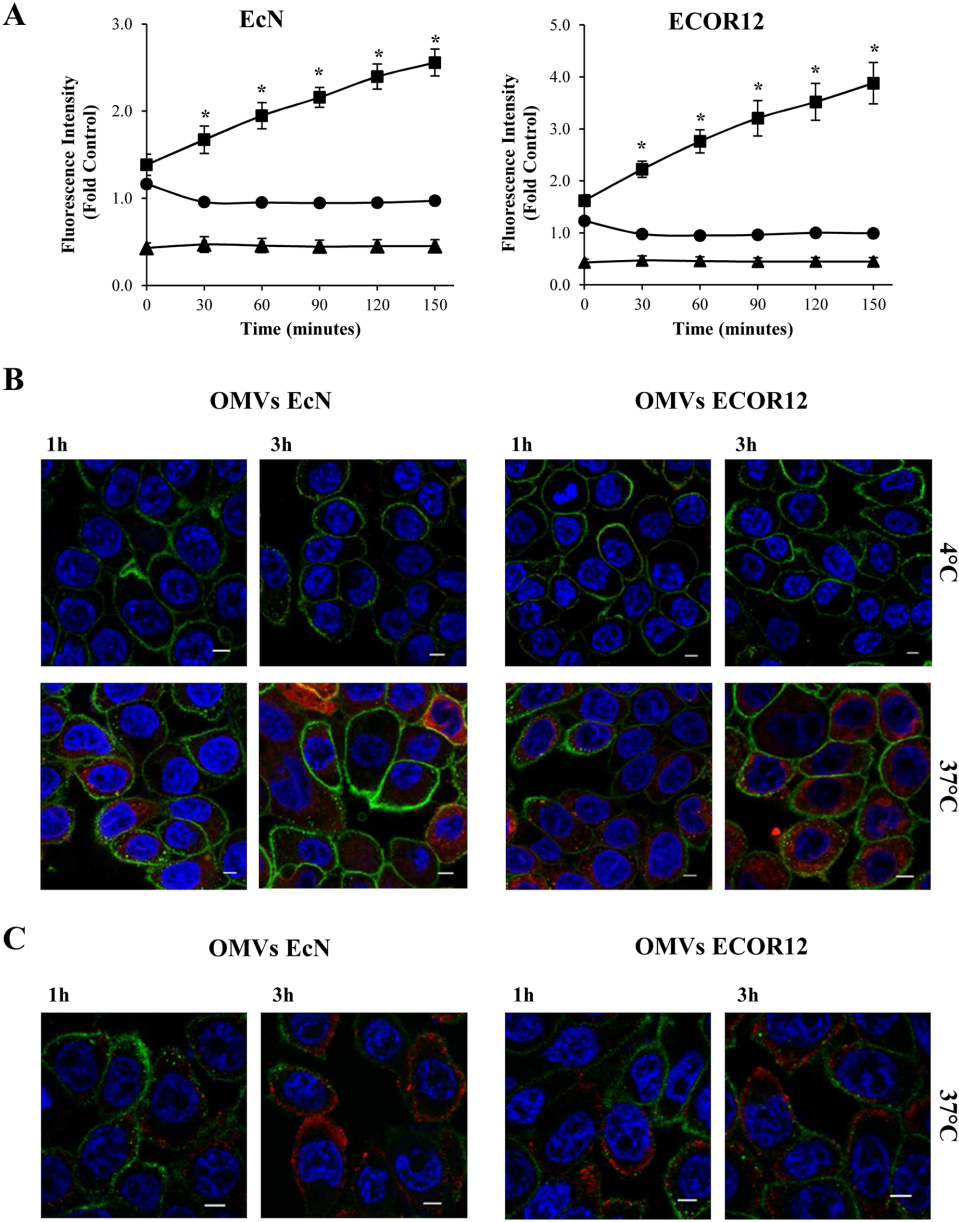
Uptake of EcN and ECOR12 OMVs in HT-29 cells. **(A)** HT-29 cells were incubated at 37°C with rhodamine B-R18-labeled OMVs (2 μg/well) from strains EcN and ECOR12 (squares) and fluorescence was measured over time with a microplate reader. OMVs (circles) and cells (triangles) alone were analyzed in parallel as controls. Fluorescence intensity was normalized by fluorescence detected at the indicated time points by labeled OMVs in the absence of cells. Data are presented as means ± standard error from four independent experiments. Results significantly different from that of untreated control cells are indicated by an asterisk (*P*<0.006). **(B, C)** Visualization of internalized OMVs by florescence microscopy. HT-29 cells were incubated with rhodamine B-R18-labeled OMVs **(B)** or with unlabeled OMVs **(C)** for 1 and 3 h at 37°C. When indicated incubations were performed at 4°C as a control. The cell membrane was stained with WGA (green) and nuclei with DAPI (blue). Internalized rhodamine B-R18-labeled OMVs are visualized in red. In **(C)** vesicles were immunostained with *E*. *coli* anti-LPS antibody and Alexa Fluor 546-conjugated secondary antibody. Analysis was performed in a Leica TCS SP5 laser scanning confocal spectral microscope with 63x oil immersion objective lens, and images were captured with a Nikon color camera (16 bit). Scale bar: 20 μm.

EcN and ECOR12 OMVs were isolated from LB cultures as described previously [28]. The absence of cell debris was assessed by electron microscopy [28,29]. We have previously shown that vesicles from both strains displayed similar protein profiles and LPS amount [29]. To study the entry mechanism of OMVs into human intestinal epithelial cells, HT-29 cells grown to 80% confluency were incubated with rhodamine B-R18-labeled OMVs (2 μg/well), and the kinetics of OMVs uptake were monitored over time using a microplate fluorescence reader. For both EcN and ECOR12 vesicles, the results showed a time-dependent increase in fluorescence, which was consistent with OMVs internalization. The fluorescence level of control samples containing only HT-29 cells or rhodamine B-R18- labeled OMVs did not increase above the background levels (Fig 1A).

OMVs internalization by HT-29 cells was confirmed by confocal fluorescence microscopy at 1 and 3 h incubation with rhodamine B-R18-labeled EcN OMVs or ECOR12 OMVs (2 μg/well). Nuclei were labeled with DAPI and plasma membranes with WGA. As expected, no red signal was observed in non-treated control cells. Rhodamine B-R18-labeled OMVs were visualized in the cytoplasm of HT-29 cells incubated at 37°C. In contrast, vesicle uptake was not observed when incubation was carried out at 4°C (Fig 1B). The intracellular location of OMVs was confirmed through experiments performed with HT-29 cells incubated with unlabeled OMVs (2 μg/well) at 37°C, followed by vesicle immunostaining with anti-*E*. *coli* LPS and Alexa Fluor 546-conjugated anti-mouse IgG antibodies (Fig 1C). Taken together, these results showed that both EcN OMVs and ECOR12 OMVs were internalized by HT-29 cells, and that the entry process was temperature-dependent.

### EcN and ECOR12 OMVs are internalized by clathrin-mediated endocytosis

**Fig 2:**
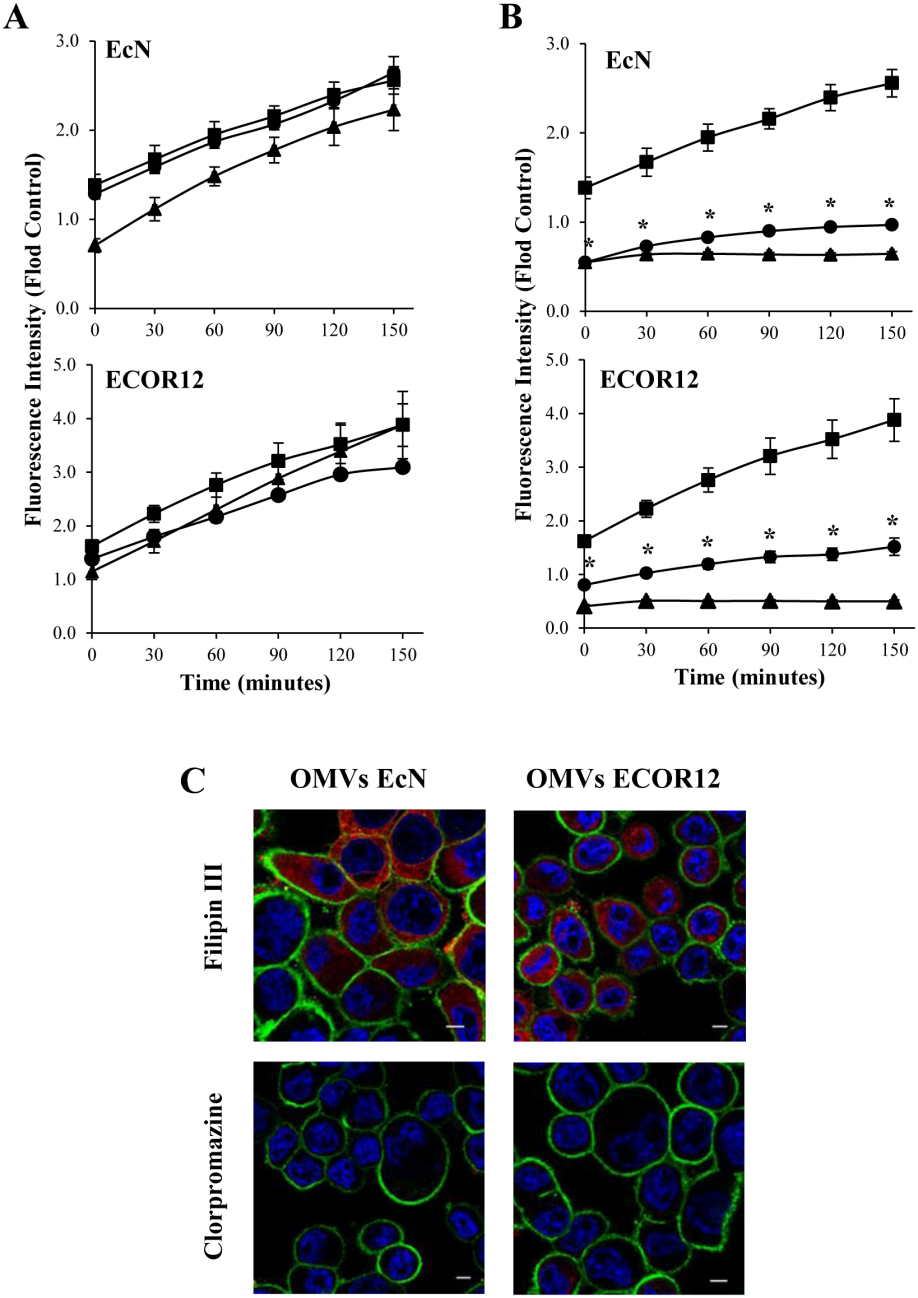
Inhibitors of dymanin and clathrin-mediated endocytosis block internalization of EcN and ECOR12 vesicles. HT-29 cells were pre-incubated for 1h at 37°C with **(A)** the lipid raft disrupting agents filipin III (triangles) or nystatin (circles), or with **(B)** the inhibitors of the clathrin-mediated endocytosis pathway chlorpromazine (circles) or dynasore (triangles) before adding rhodamine B-R18-labeled OMVs (2 μg/well) from strains EcN and ECOR12. Uptake experiments were performed in HT-29 cells in the absence of endocytosis inhibitors for comparison (squares). Fluorescence was measured over time with a microplate reader. Fluorescence intensity was normalized by fluorescence detected at the indicated time points by labeled OMVs in the absence of cells. Data are presented as means ± standard error from four independent experiments. Results significantly different from that of cells incubated with OMVs in the absence of endocytosis inhibitors are indicated by an asterisk (*P*<0.012). **(C)** Analysis by fluorescence microscopy of vesicle uptake in the presence of endocytosis inhibitors. HT-29 cells were pre-incubated with chlorpromazine or filipin III for 1 h before the addition of rhodamine B-R18-labeled OMVs (2 μg). After 1 h incubation, the cell membrane was stained with WGA (green) and nuclei with DAPI (blue). Internalized OMVs are visualized in red. Images are from a single representative experiment (n = 4). Scale bar: 20 μm.

**Fig 3:**
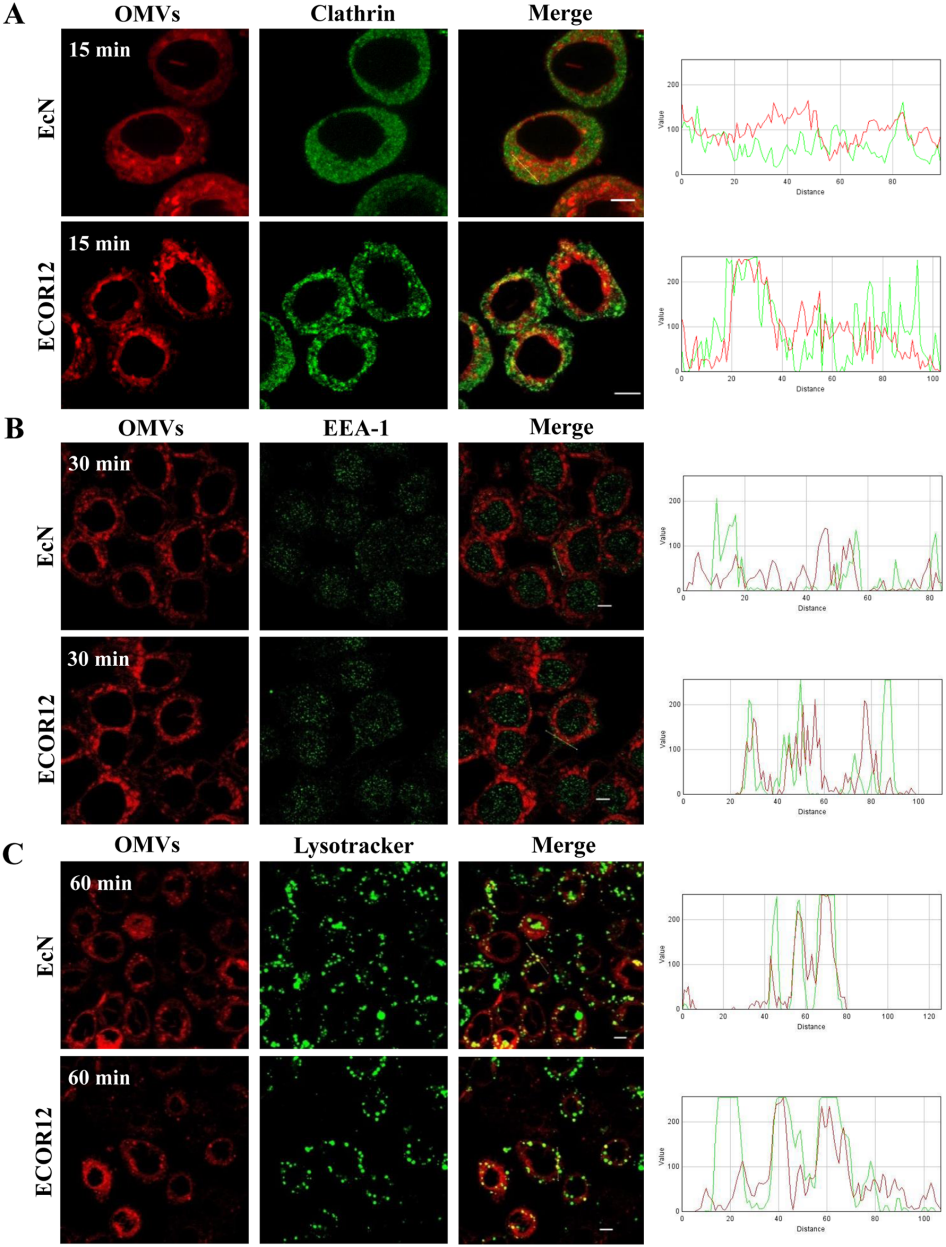
Colocalization of EcN and ECOR12 OMVs with clathrin (A), endosomes (B) and lysosomes (C). HT-29 cells were incubated with rhodamine B-R18-labeled OMVs (2 μg) for the indicated times and analyzed using laser scanning confocal spectral microscope. Scale bar: 20 μm. Clathrin was stained using anti-clathrin mouse monoclonal antibody and Alexa Fluor 488-conjugated goat anti-mouse IgG (green). Endosomes were labeled with a rabbit polyclonal antibody against the endosome-associated protein EEA1 and Alexa Fluor 488-conjugated goat anti-rabbit IgG (green). Lysosomes were detected using LysoTracker Green DND-26 at 300 nM (green). Images are from a single representative experiment (n = 4). Colocalization of the green (clathrin, EEA1 or the LysoTracker probe)) and red (vesicles) signals was confirmed by histogram analysis of the fluorescence intensities along the yellow lines. Analysis by laser scanning confocal spectral microscope was performed as described for Fig 1.

The internalization pathway used by bacterial OMVs depends on the strain. Vesicles from *E*. *coli* pathogens can enter host cells by clathrin-dependent endocytosis or via lipid rafts [13,14]. To identify the endocytic pathway involved in the entry of EcN and ECOR12 OMVs, time-course internalization experiments using rhodamine B-R18-labeled OMVs were performed in the presence of various inhibitors of endocytosis. OMVs entry was assessed by monitoring the increase in fluorescence emitted by HT-29 cells upon OMVs addition. Disruption of lipid rafts microdomains and caveolae by treatment with the cholesterol-sequestering agent’s filipin III or nystatin did not significantly reduce OMVs internalization by HT-29 cells (Fig 2A). This indicated that cholesterol-enriched lipid rafts are not required for the entry process. In contrast, vesicle internalization was drastically inhibited in chlorpromazine- or dynasore treated-cells (*p*>0.012) (Fig 2B). Chlorpromazine is an inhibitor of clathrin-dependent endocytosis [33] and dynasore inhibits dynamin, a protein essential in membrane remodelling and fission of clathrin-coated vesicles [34]. The effect of endocytosis inhibitors on microbiota OMVs internalization was confirmed by microscopy analysis. As shown in Fig 2C, internalization of rhodamine B-R18-labeled OMVs in HT-29 cells was specifically inhibited by chlorpromazine, whereas filipin III had no effect. Overall, these data indicate that OMVs from probiotic and commensal *E*.*coli* strains are internalized in HT-29 cells via clathrin-mediated endocytosis. The contribution of this endocytic pathway was further assessed by analysing colocalization of EcN and ECOR12 OMVs with clathrin in HT-29 cells. Cells were incubated for 15 min with rhodamine B-R18-labeled OMVs, or with transferrin-Alexa Fluor 633 as a positive control, before immunostaining with anti-clathrin and Alexa Fluor 488-conjugated secondary antibodies. Colocalization of rhodamine B-R18-labeled vesicles with clathrin was visualized by fluorescence microscopy as yellow spots in the merged images (Fig 3A). To measure colocalization of these vesicles with clathrin, the overlap coefficients (r) were calculated from data collected from four independent experiments. This analysis yielded r values of 0.71±0.07 for EcN OMVs and 0.603±0.025 for ECOR12 OMVs. These coefficients were similar to that of the positive control transferrin (r = 0.556±0.028). Images showing colocalization of transferrin and clathrin are presented as supporting information (S1 Fig).

We next tested whether this endocytic pathway mediates OMVs entry in other human intestinal epithelial lines, such as Caco-2 and HT29-MTX cells. Uptake experiments with rhodamine B-R18-labeled OMVs were performed in the absence or presence of endocytosis inhibitors (chlorpromazine and filipin III) in Caco-2 cells grown to 80% confluency and in differentiated Caco-2 and HT29-MTX cells (17 days post-confluence). Upon the addition of labeled OMVs (2 μg/well), fluorescence was recorded over 150 min with a plate reader. Data showed that internalization of EcN and ECOR12 OMVs by polarized epithelial cell monolayers also takes place through the clathrin-dependent endocytosis pathway (S2 Fig). In the absence of specific endocytic inhibitors, the time-dependent increase in fluorescence detected in differentiated cultures of the mucin-producer HT29-MTX cell line indicated that bacterial OMVs can diffuse through the mucus layer before their entry into target cells.

### EcN and ECOR12 OMVs are sorted to lysosomal compartments

Intracellular trafficking of microbiota *E*. *coli* OMVs was assessed by monitoring vesicle association with endocytic compartments, specifically with early endosomes and lysosomes. We found that at 30 min incubation, rhodamine B-R18-labeled OMVs colocalized with the endosome-associated EEA1 protein in HT-29 cells (Fig 3B). The degree of colocalization was estimated from the corresponding overlap coefficients, with values of 0.422±0.071 for EcN and 0.366±0.017 for ECOR12 vesicles. The location of OMVs into early endosomes was confirmed by vesicle immunostaining with anti-*E*. *coli* LPS antibodies (S3 Fig), and quantitative colocalization analysis from the collected images (r = 0.440±0.08 for EcN and r = 0.39±0.045 for ECOR12 vesicles). At 30 and 60 min, OMVs were also detected into lysosomal compartments, as shown by their colocalization with LysoTracker Green DND-26 (Fig 3C). At 30 min, colocalization was also evident (not shown). At 60 min, the overlap coefficients were 0.546±0.031 for EcN and 0.481±0.005 for ECOR12 vesicles. Time course analyses from 15 min to 5 h did not reveal any vesicle-derived fluorescence signal in the cell nuclei.

### EcN and ECOR12 OMVs inhibit HT-29 cell growth without affecting cell viability

**Fig 4:**
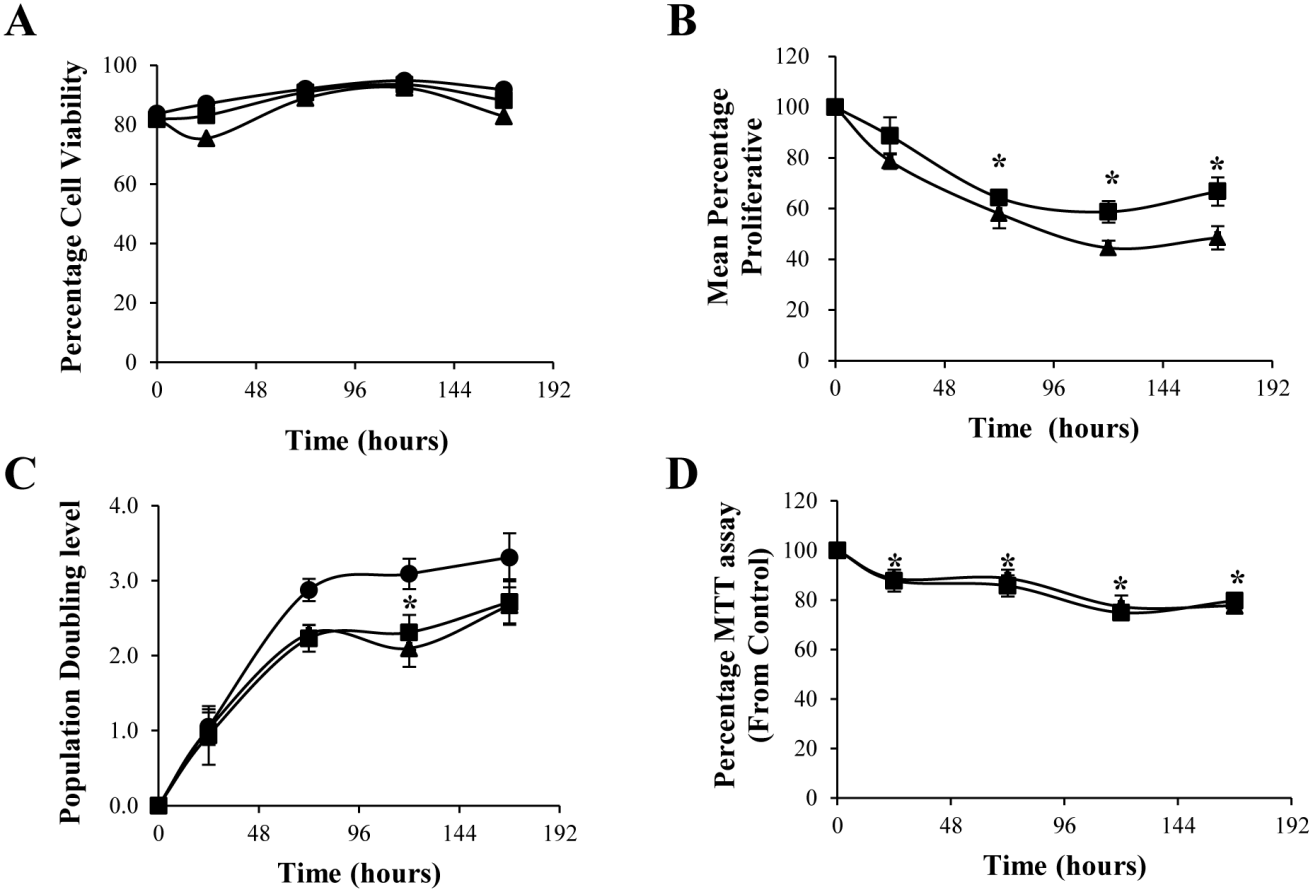
Effect of EcN and ECOR12 OMVs on proliferation and viability of HT-29 cells. **(A)** Cell viability, **(B)** Mean proliferation index, **(C)** Population doubling level of HT-29 cells exposed to OMVs (5 μg/ml) from EcN (squares) or ECOR12 (triangles) for up to 7 days, measured by the trypan blue exclusion assay. In panel **(C)**, control cells are indicated by circles. **(D)** MTT reduction activity measured along the experiment. Values are means ± standard error from four independent experiments (*P*<0.01, versus untreated control cells).

Since OMVs from some pathogenic *E*. *coli* strains induce proliferation of Caco-2 cells, we examined the impact of gut microbiota-derived OMVs on the cell growth of the intestinal epithelial cell line HT-29. To this end, kinetic studies were performed in HT-29 cells exposed to EcN or ECOR12 OMVs (5 μg/ml) for up to 168 h. Cell numbers were calculated by trypan blue exclusion assays carried out every second day during the experiment. Results showed that cell viability was not significantly altered by OMVs treatment. The number of dead cells did not increase upon incubation with microbiota *E*. *coli* vesicles (Fig 4A). HT-29 cells grew exponentially up to 72 h, and then the growth rate began to decline as they reached confluency. The growth of cells treated with either EcN or ECOR12 OMVs was lower than that of unstimulated control cells (Fig 4B). Growth reduction values were statistically significant from 72 h (around 50% and 60% respectively compared to control cells, p<0.01) until the end of the experiment. Consistently, the population doubling levels were lower in OMVs-treated cells than in control cells (Fig 4C). The calculated PDT values were 17.42±1.70 h for untreated control cells, 30±1.40 h for cells incubated with EcN OMVs and 25.76±1.15 h for cells incubated with ECOR12 OMVs.

**Fig 5:**
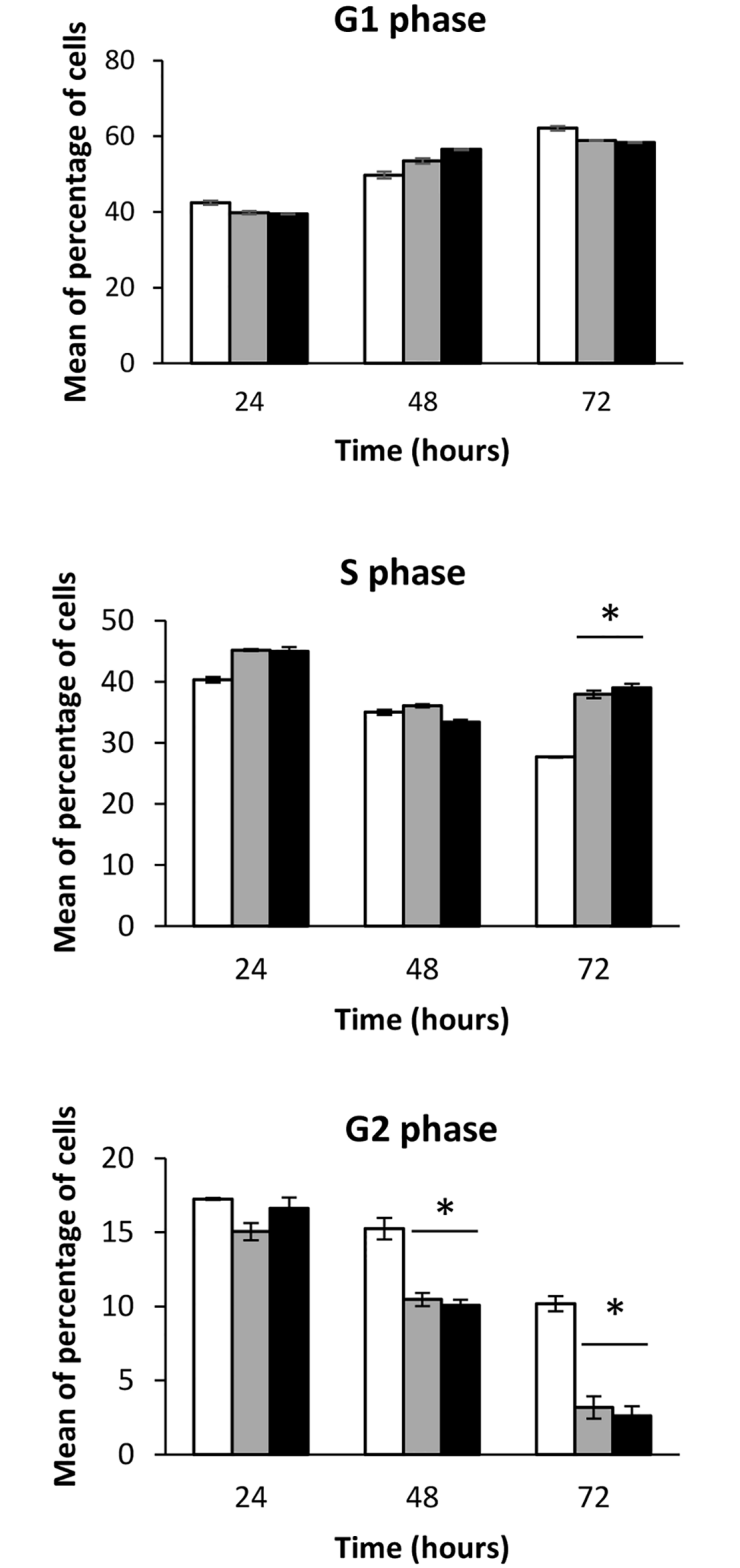
EcN and ECOR12 OMVs induce cell cycle arrest in HT-29 cells. Cell cycle analysis of HT-29 cells challenged with OMVs (5 μg/ml) from strains EcN (gray bars) or ECOR12 (black bars) for up to 3 days. Non-treated cells were analyzed as a control (white bars). Cells were gated based on FSC and SSC and then by their area *vs* peak fluorescence signal for propidium iodide. A total of 10,000 events were analyzed for each sample. Values are means ± standard error from three independent experiments (*P*<0.02, versus untreated control cells).

In addition to the trypan blue assay, viable cells were also estimated using MTT (Fig 4D). Results showed around 20% lower MTT reduction levels in cells treated with OMVs than in untreated control cells. These data were consistent with the reduction in the growth rates, and hence in the cell number, deduced from the trypan blue assays. Overall, these results suggested that EcN and ECOR12 OMVs inhibit cell proliferation, but have no cytotoxic effects on intestinal epithelial cells. We next examined the effect of the bacterial vesicles on cell cycle progress in HT-29 cells incubated with OMVs (5 μg/ml) for 24, 48 and 72 h (Fig 5). Flow cytometry analysis of cells labeled with propidium iodide showed no significant differences in the percentage of cells in the G1 phase between control and treated cells. However, microbiota *E*. *coli* OMVs increased the percentage of cells in the S phase, with values that were statistically significant at 72 h incubation. Consistent with this finding, the number of cells reaching the G2 phase was significantly lower upon 48 and 72 h incubation in cells challenged with OMVs than in non-treated cells (*P*<0.02). These results indicated that OMVs promote S/G2 cell cycle arrest in HT-29 cells. This effect may account for the observed OMVs-inhibition effect on cell growth.

### DNA damage and repair induced by EcN and ECOR12 OMVs

**Fig 6:**
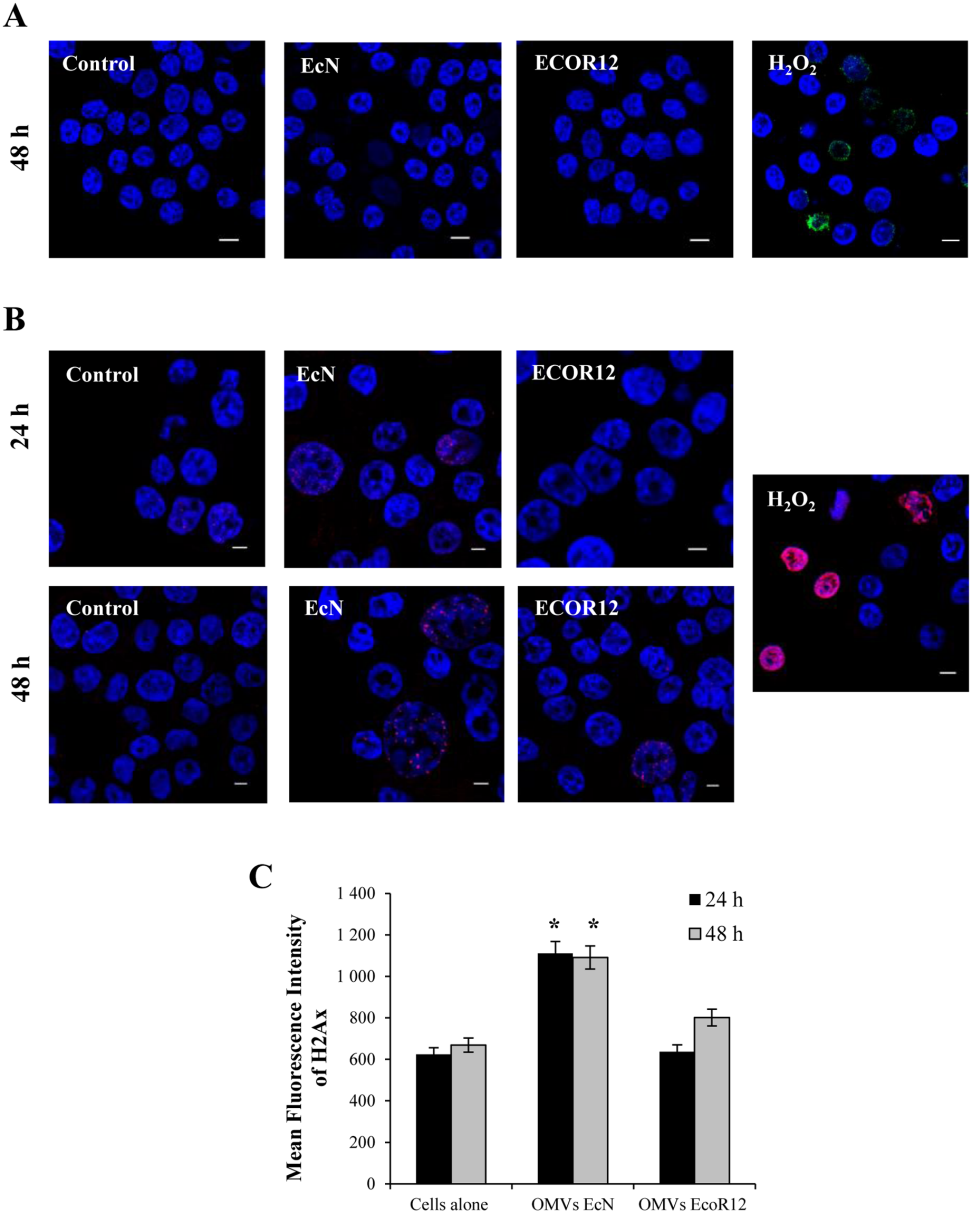
DNA damage analysis in HT-29 cells exposed to EcN or ECOR12 OMVs. **(A)** Immunofluorescence microscopy of mutagenic 8-oxo-dG adducts (green) in cell nuclei of cells challenged with the indicated OMVs (5 μg/ml) for 48 h. **(B)** Immunofluorescence microscopy of phosphorylated γH2AX (red) in cell nuclei of cells challenged with the indicated OMVs (5 μg/ml). Cells treated with 300 μM H_2_O_2_ for 24 h were processed in parallel as a positive control. Immunofluorescence microscopy images are from a single representative experiment (n = 3). **(C)** Flow cytometry analysis of phosphorylated γH2AX expression. Cells were gated using the FSC *vs* SSC double dot. A total of 10,000 events were analyzed for each sample. Data are presented as means ± standard error from three independent experiments. Results significantly different from that of untreated control cells are indicated by an asterisk (*P*<0.02).

OMVs from some *E*. *coli* strains induce oxidative stress in intestinal epithelial cells, leading to the formation of DNA lesions, such as 8-oxo-dG adducts [10]. To examine whether EcN and ECOR12 derived OMVs induce these kind of DNA lesions, immunofluorescence microscopy analysis was used to detect 8-oxo-dG adducts in HT-29 cells exposed to OMVs (5 μg/ml) for up to 7 days. As a control, cells were challenged with 300 μM H_2_O_2_ for 24 h (Fig 6A). Whereas 8-oxo-dG staining was apparent in the nuclei of cells treated with H_2_O_2_, no fluorescent signal attributable to 8-oxo-dG was visible in either untreated or OMVs-treated cells throughout the experiment. These results suggested that EcN and ECOR12 OMVs do not induce oxidative stress in gut epithelial cells.

Some bacterial toxins present in OMVs from *E*.*coli* O157:H7 strains can produce DSBs in DNA [10]. These DNA lesions are also produced by *E*. *coli* strains harbouring the *pks* gene island responsible for the synthesis of the genotoxic polyketide colibactin [35]. The probiotic strain EcN is among the colibactin-producing strains. It has been shown that EcN causes high levels of DNA DSBs in intestinal epithelial cells, whereas a derived *ΔclbA* mutant, impaired in colibactin synthesis, fails to do so. These experiments were performed in cultured intestinal cells infected with bacterial suspensions [36]. Based on this information, we sought to analyse whether OMVs from this probiotic strain have the potential to induce this kind of DNA damage. Vesicles from the commensal strain ECOR12 were also included for comparison. As the repair of DSBs in DNA requires recruitment and phosphorylation of the histone γH2AX to the damaged sites, immunostaining of phosphorylated γH2AX was performed in HT29 cells after 24 and 48 h incubation with EcN or ECOR12 OMVs (Fig 6B). Untreated control cells displayed a very low level of punctuate red nuclear staining in fluorescence micrographs, which is compatible with free radical generation of DNA damage and repair in normal dividing cells. However, following exposure to EcN OMVs, considerably more cells displayed the red nuclear staining either at 24 h or 48 h (22.5% positive nuclei versus 13.6% in untreated cells). In this case, labeled nuclei were enlarged. In cells challenged with ECOR12 OMVs, immunolabeling of phosphorylated γH2AX was only apparent in some cells after 48 h treatment (14.5% positive nuclei versus 13.6% in untreated cells). These results were from three independent experiments, and for each condition the total number of cells analysed ranged in between 150–200. Quantification of phosphorylated γH2AX by flow cytometry confirmed the immunofluorescence microscopy results (Fig 6C). A significant increase in fluorescence intensity with respect to unstimulated HT-29 cells was observed upon exposure to EcN OMVs (*P*<0.02), whereas vesicles from the commensal ECOR12 strain only promoted a slight increase in fluorescence intensity at 48 h, which was not statistically significant. Overall, these results suggested that probiotic EcN OMVs induced a larger proportion of DSBs, and therefore more DNA damage, than ECOR12 OMVs. Consistent with these results, alkaline single-cell electrophoresis analysis revealed longer DNA tails in cells exposed to EcN OMVs (S3 Fig).

## Discussion

It is well-stablished that OMVs are a mechanism used by pathogens to secrete and deliver bacterial virulence or immunomodulatory factors into host cells. Released OMVs allow direct interaction of bacterial components with cellular receptors before the whole bacterium interacts with or enters mammalian target cells. Therefore, this delivery system provides injury and immune subversion mechanisms to facilitate infection. OMVs from Gram-negative pathogens are mainly internalized by host cells via endocytosis [13–16]. The uptake pathway depends on particular vesicular proteins or components that target these vesicles to specific cellular receptors or lipid membrane microdomains. This has been shown for the OMVs released by enterotoxigenic *E*. *coli*, in which the heat-labile enterotoxin LT interacts with its specific cell surface receptor GM1, targeting OMVs to lipid raft-mediated endocytosis [14]. In contrast, OMVs from EHEC enter epithelial cells via CME. In this case, the toxin hemolysin is delivered and targeted to mitochondria through OMVs, but it is not required for the endocytic process [13].

Although the number of studies dealing with gut microbiota OMVs is still limited, bacterial vesicles are emerging as key players in the communication between microbiota and intestinal mucosa cells. A proteomic analysis of OMVs released by the probiotic EcN revealed that, in addition to strain-specific proteins, these vesicles contain sets of proteins also found in the OMVs generated by Gram-negative pathogens that provide essential functions for the establishment and persistence of bacteria (either pathogens or probiotics) in the host tissues [28]. Examples of these proteins are antimicrobial activities to kill competing bacteria, surface-associated proteins that promote adhesion to host tissues, and factors that can modulate the host immune response. As the uptake pathway for gut microbiota vesicles has not been reported so far, we approached internalization studies in intestinal epithelial cell lines using as a model the OMVs released by two *E*. *coli* strains that colonize the human gut, the probiotic strain EcN and the commensal strain ECOR12. These strains differ in their bacterial surface, as EcN expresses a capsular K5 polysaccharide that is widespread in some pathogenic bacterial groups and mediates interactions with intestinal epithelial cells [37]. In spite of this difference, OMVs from both strains entered epithelial cells via CME, as vesicle uptake was clearly blocked by chlorpromazine and dynasore. Dynasore was first described as a dynamin inhibitor, thus interfering with the remodeling and fission of clathrin-coated vesicles during endocytosis. Dynasore also has other effects that are dynamin-independent, such as its ability to disrupt lipid raft organization by reducing labile cholesterol in the plasma membrane [38]. Since, filipin III and nystatin, both cholesterol-sequestering agents, do not impair internalization of EcN and ECOR12 OMVs, the effect of dynasore on the uptake of these microbiota vesicles must be attributed to CME inhibition. In polarized epithelia, uptake of these microbiota vesicles follows the same CME pathway. In addition, our results in the mucin-producer HT29-MTX cell line show that OMVs can diffuse through the mucus layer before their entry into target cells. Following the CME pathway, these vesicles are sorted to endocytic compartments, reaching lysosomes. Colocalization studies evidenced the presence of these OMVs in early endosomes and lysosomes. The overlap coefficients (higher than or close to 0.5) confirmed colocalization of EcN and ECOR12 with specific markers of these endocytic compartments. The overlap coefficients calculated for the colocalization with EEA1 were slightly below 0.5, which can be explained by the transient location of vesicles in early endosomes during their trafficking to lysosomes. Once these vesicles reach early endosomes they can activate NOD1-signalling responses through specific binding of peptidoglycan to this intracellular receptor [39,40]. In the acidic lysosomes, some specific protein cargo in OMVs may be separated or dissociated from the whole vesicle inside this endocytic compartment, and then targeted to other organelles or subcellular locations to induce particular responses. This is, for instance, the fate of EHEC hemolysin, which is separated from OMVs after endosomal acidification, and is targeted to mitochondria to cause apoptosis [13].

Considering our results, although OMVs have not been observed in the nuclei, EcN vesicles promote DSB in eukaryotic cell DNA, as shown by the immunoquantification of phosphorylated γH2AX and the formation of DNA tails in the Comet assay. In this probiotic strain, genotoxicity has been attributed to colibactin [36]. This toxin is a non-ribosomal peptide-polyketide, synthesized by enzyme activities encoded in the *pks* island, which belongs to a largely uncharacterized family of small genotoxic molecules that induce DSB in DNA. Studies performed with an EcN mutant deficient in colibactin synthesis proved that, in addition to its genotoxic activity, colibactin is also required for the *in vivo* anti-inflammatory effects of this probiotic. Moreover, deficiency in colibactin biosynthesis leads to the exacerbation of colitis severity in dextran sulfate sodium-treated mice [36]. These facts lead authors to considered colibactin as an immunomodulin. Interestingly, the probiotic activity of EcN cannot be dissociated from its genotoxicity, and both effects require a functional colibactin biosynthetic pathway [36]. How this toxin is exported and delivered into the host infected cell remains unknown. As EcN OMVs induce the same type of DNA lesions as colibactin, we may speculate that colibactin or intermediate metabolites of colibactin biosynthesis could be delivered to mammalian cells by OMVs. In contrast, OMVs from the commensal strain ECOR12 (*pks* negative) display low ability to induce DSB lesions. Although the protein profile and LPS content of EcN and ECOR12 vesicles are very similar [29], these strains belong to a different phylogenetic group and therefore differ in their genomic profiles. These genomic differences may impact on the proteomic content of the released vesicles. Therefore, contribution to genotoxicity of EcN-specific vesicular components other than colibactin cannot be ruled out.

In general, *E*. *coli* OMVs may be genotoxic to human intestinal epithelial cells. Even vesicles released by the avirulent *E*. *coli* strain DH5α induce the generation of radical oxygen species that lead to DNA damage and the formation of mutagenic 8-oxo-dG adducts [10]. Our results show that neither the probiotic EcN nor the commensal ECOR12 OMVs induce this kind of oxidative lesions in the DNA of epithelial cells upon internalization. It is known that accumulation of DNA damage results in cell cycle arrest before DNA is repaired. Analysis of the cell cycle progression indicates that both EcN and ECOR12 OMVs promote S/G2 cell cycle arrest in HT-29 cells. These results are compatible with the p53 mutation present in this cell line. Deficiency in p53 abolishes the G1 checkpoint, which is essential to control the entry of cells in the S phase.

OMVs from the gut microbiota strains EcN and ECOR12 do not affect cell viability, but reduce the proliferation rate. These effects are clearly different from what has been reported for some *E*. *coli* pathogenic strains. Regarding cell viability, specific toxins present in the OMVs of EHEC induce apoptosis and cell death [11,13]. Consistently, vesicles from the non-pathogenic *E*. *coli* strain DH5a do not alter cell viability nor affect cell growth rates [10]. Considering cell growth, OMVs from EHEC or adherent-invasive *E*. *coli* significantly increase cell proliferation [10]. This fact together with their genotoxic-associated activity led to the hypothesis that, like *Helicobacter pylori* vesicles [41,42], OMVs from these *E*. *coli* strains may potentially influence the development of cancer [10].

## Conclusions

To our knowledge this is the first study exploring the internalization pathway used by membrane vesicles released by gut microbiota strains to enter intestinal epithelial cells. In conclusion, the OMVs produced by the *E*. *coli* probiotic EcN and the commensal ECOR12 strains are internalized by epithelial cells through CME, and sorted to lysosomes via endocytic compartments. During intracellular trafficking, these vesicles could activate intracellular NOD receptors to induce cellular responses. These vesicles do not affect cell viability nor cause oxidative damage on DNA. OMVs from the probiotic strain EcN specifically cause DSBs in DNA, which are repaired through recruitment of phosphorylated γH2AX. Both the genotoxic and anti-inflammatory effects of EcN have been attributed to colibactin [36]. Whether this immunomodulin is exported and delivered into the human gut by OMVs is an open question that needs further study.

## Supporting Information

S1 Fig Colocalization analysis of chlatrin and transferrin in fluorescence microscopy. Immunostaining of clathrin (green) was carried out after 15 min incubation of HT-29 cells with transferrin-Alexa Fluor 633 (red). Colocalized green and red signals appear in yellow. Scale bar: 20 μm. Colocalization of the green (clathrin) and red (transferrin) signals was assessed by histogram analysis of the fluorescence intensities along the yellow line. Images are from a single representative experiment (n = 3). Analysis was performed in a Leica TCS SP5 laser scanning confocal spectral microscope with 63x oil immersion objective lens, and images were captured with a Nikon color camera (16 bit).
(TIF)

S2 Fig Analysis of OMVs internalization by polarized Caco-2 or HT29-MTX cells in the presence of endocytosis inhibitors. Confluent monolayers of fully polarized Caco-2 **(A)** or HT29-MTX **(B)** cells (17 days post-confluence) were pre-incubated for 1h at 37°C with filipin III (triangles) or chlorpromazine (circles) before adding rhodamine B-R18-labeled OMVs (2 μg/well) from strains EcN and ECOR12. Uptake experiments were performed in the absence of the endocytosis inhibitors for comparison (squares). Fluorescence was measured over time with a microplate reader. Fluorescence intensity was normalized by fluorescence detected at the indicated time points by labeled OMVs in the absence of cells. Data are presented as means ± standard error from three independent experiments. Results significantly different from that of cells incubated with OMVs in the absence of endocytosis inhibitors are indicated by an asterisk (*P*<0.03).
(TIF)

S3 Fig Colocalization analysis of EEA- with EcN and ECOR12 vesicles in fluorescence microscopy. Analysis was carried out after 30 min incubation of HT-29 cells with OMVs (2 μg) from the indicated strains. Early endosomes were immunostained with a rabbit polyclonal antibody against the endosome-associated protein EEA1 and Alexa Fluor 488-conjugated goat anti-rabbit IgG (green). Internalized vesicles were immunostained with *E*. *coli* anti-LPS antibody and Alexa Fluor 546-conjugated secondary antibody (red). Colocalized green and red signals appear in yellow. Scale bar: 20 μm. Colocalization of the green (EEA1) and red (vesicles) signals was assessed by histogram analysis of the fluorescence intensities along the yellow line. Images are from a single representative experiment (n = 3). Analysis by laser scanning confocal spectral microscope was performed as described in S1 Fig.
(TIF)

S4 Fig Analysis of OMV-induced DNA double strand breaks in HT-29 cells by the Comet assay. HT-29 cells treated with the indicated OMVs (5 μg/ml) for 48 h or with 300 μM H_2_O_2_ for 24 h were trypsinized and processed for alkaline cell-single electrophoresis assay. DNA was stained with ethidium bromide (20 μg/ml). The slides were examined using a Leica D1000 microscope with a 63x oil immersion objective. Images are from a single representative experiment (n = 3).
(TIF)

## Abbreviations

CME: Clathrin-mediated endocytosis
DBS: double-strand breaks
DMEM: Dulbecco’s modified Eagle medium
EcN: *Escherichia coli* Nissle 1917
FCS: fetal calf serum
FSC: forward scatter
LB: Luria-Bertani broth
MPI: mean proliferative index
MTT: 3-(4,5-dimethylthiazol-2-yl)-2,5-diphen yltetrazolium bromide
OMVs: outer membrane vesicles
8-oxo-dG: 8-oxo-7,8-dihydro-2´-deoxyguanosine
PBS: phosphate buffer saline
PDT: population doubling time
SSC: side scatter
WGA: fluorescent wheat germ agglutinin
