# Supplementary figures and images for: Outer Membrane Vesicles from the Probiotic Escherichia coli Nissle 1917 and the Commensal ECOR12 Enter Intestinal Epithelial Cells via Clathrin-Dependent Endocytosis and Elicit Differential Effects on DNA Damage
Source: PLoS One. 2016 Aug 3;11(8):e0160374. doi: 10.1371/journal.pone.0160374 (PMC4972321; doi:10.1371/journal.pone.0160374)

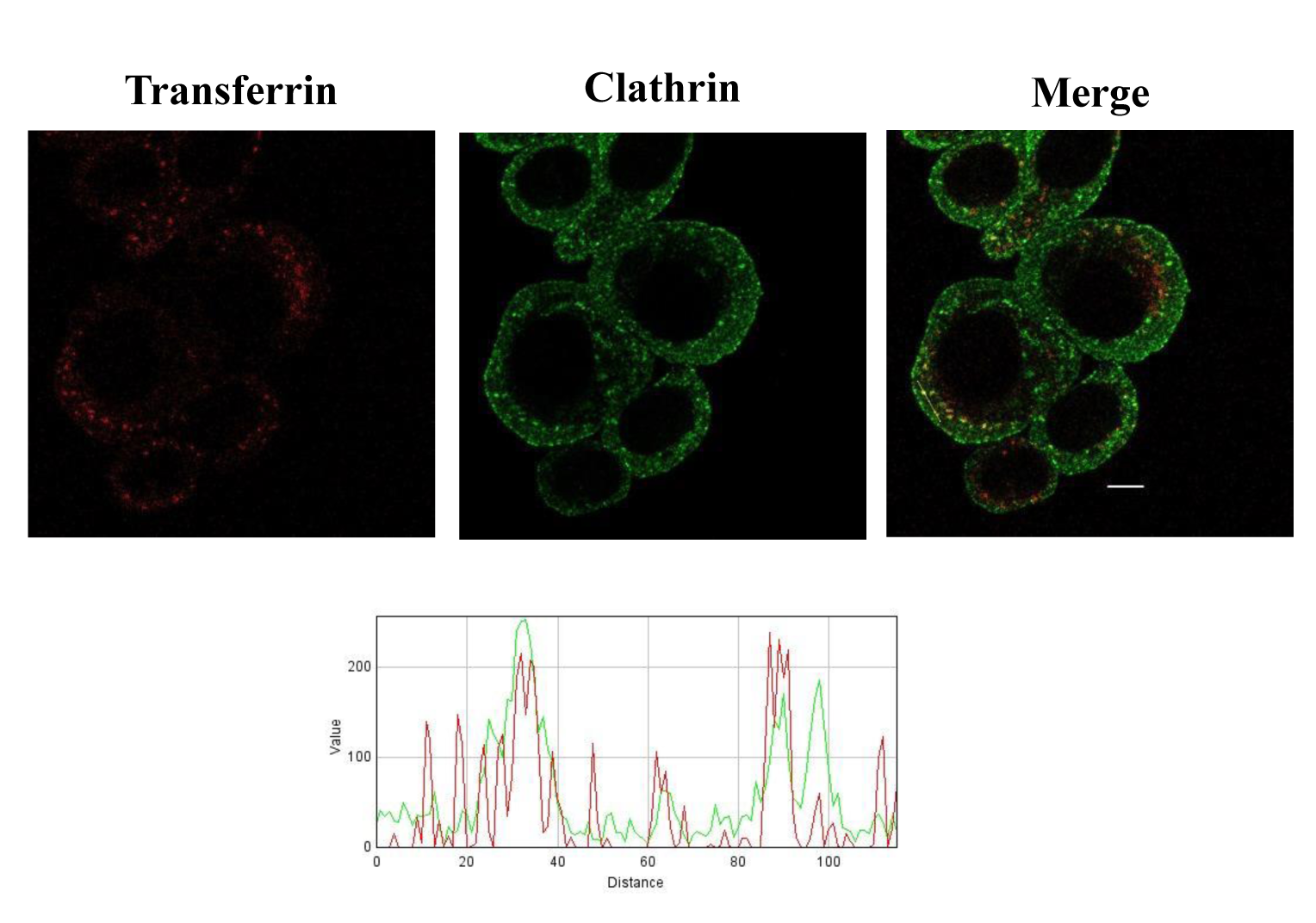

Supplement: S1 Fig — Immunostaining of clathrin (green) was carried out after 15 min incubation of HT-29 cells with transferrin-Alexa Fluor 633 (red). Colocalized green and red signals appear in yellow. Scale bar: 20 μm. Colocalization of the green (clathrin) and red (transferrin) signals was assessed by histogram analysis of the fluorescence intensities along the yellow line. Images are from a single representative experiment (n = 3). Analysis was performed in a Leica TCS SP5 laser scanning confocal spectral microscope with 63x oil immersion objective lens, and images were captured with a Nikon color camera (16 bit). (TIF) [file pone.0160374.s001.tif]

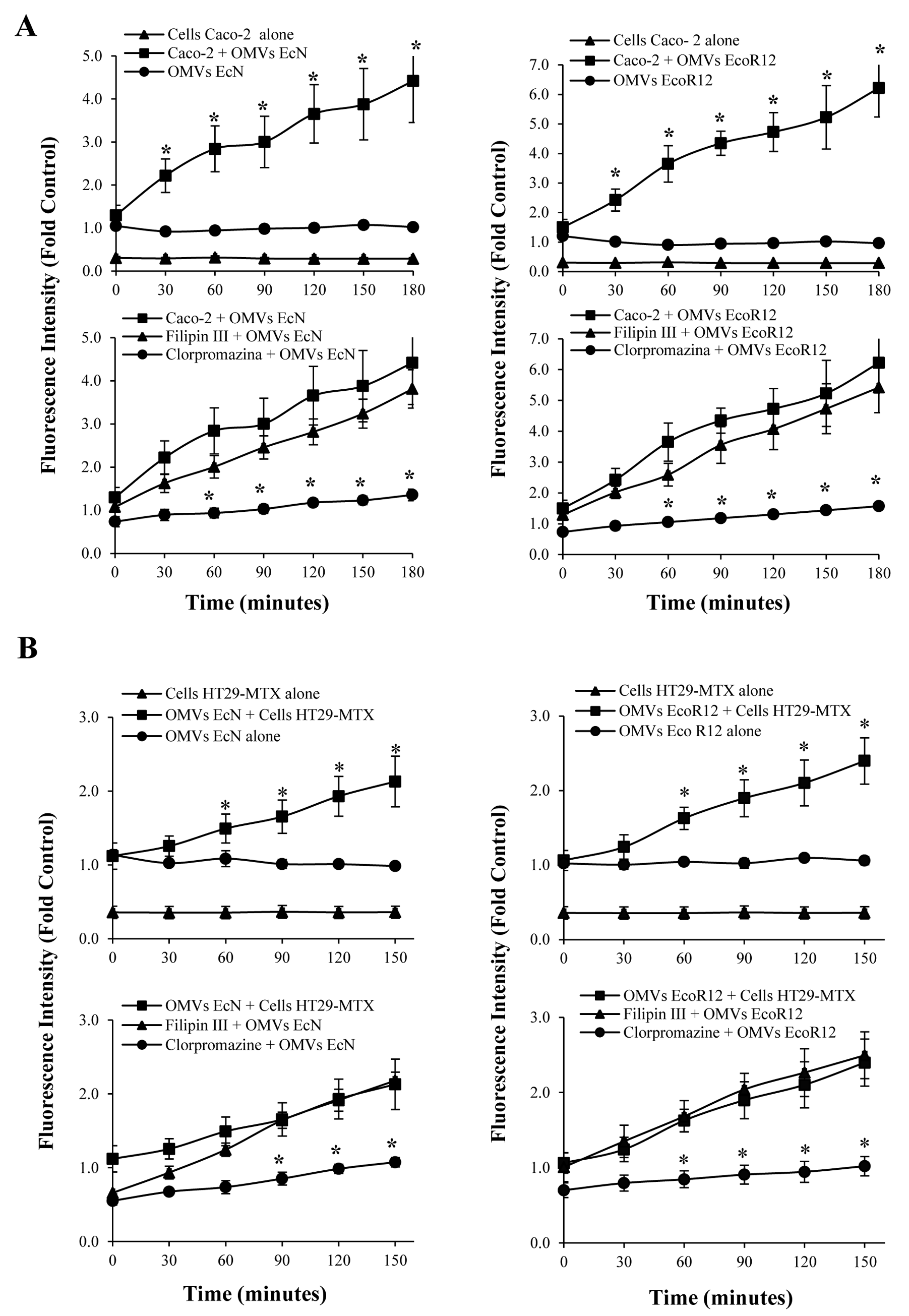

Supplement: S2 Fig — Confluent monolayers of fully polarized Caco-2 (A) or HT29-MTX (B) cells (17 days post-confluence) were pre-incubated for 1h at 37°C with filipin III (triangles) or chlorpromazine (circles) before adding rhodamine B-R18-labeled OMVs (2 μg/well) from strains EcN and ECOR12. Uptake experiments were performed in the absence of the endocytosis inhibitors for comparison (squares). Fluorescence was measured over time with a microplate reader. Fluorescence intensity was normalized by fluorescence detected at the indicated time points by labeled OMVs in the absence of cells. Data are presented as means ± standard error from three independent experiments. Results significantly different from that of cells incubated with OMVs in the absence of endocytosis inhibitors are indicated by an asterisk (P<0.03). (TIF) [file pone.0160374.s002.tif]

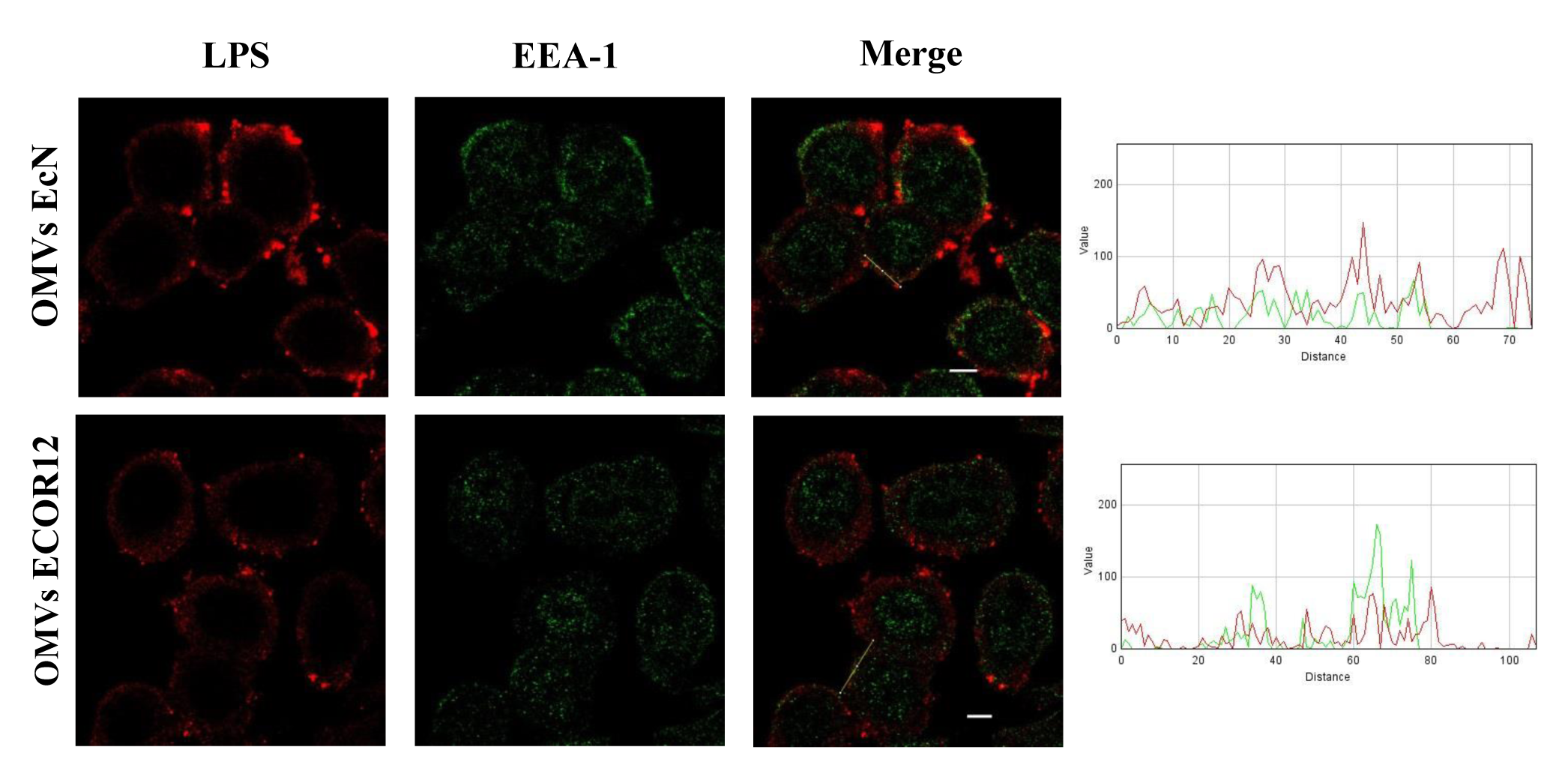

Supplement: S3 Fig — Analysis was carried out after 30 min incubation of HT-29 cells with OMVs (2 μg) from the indicated strains. Early endosomes were immunostained with a rabbit polyclonal antibody against the endosome-associated protein EEA1 and Alexa Fluor 488-conjugated goat anti-rabbit IgG (green). Internalized vesicles were immunostained with E. coli anti-LPS antibody and Alexa Fluor 546-conjugated secondary antibody (red). Colocalized green and red signals appear in yellow. Scale bar: 20 μm. Colocalization of the green (EEA1) and red (vesicles) signals was assessed by histogram analysis of the fluorescence intensities along the yellow line. Images are from a single representative experiment (n = 3). Analysis by laser scanning confocal spectral microscope was performed as described in S1 Fig. (TIF) [file pone.0160374.s003.tif]

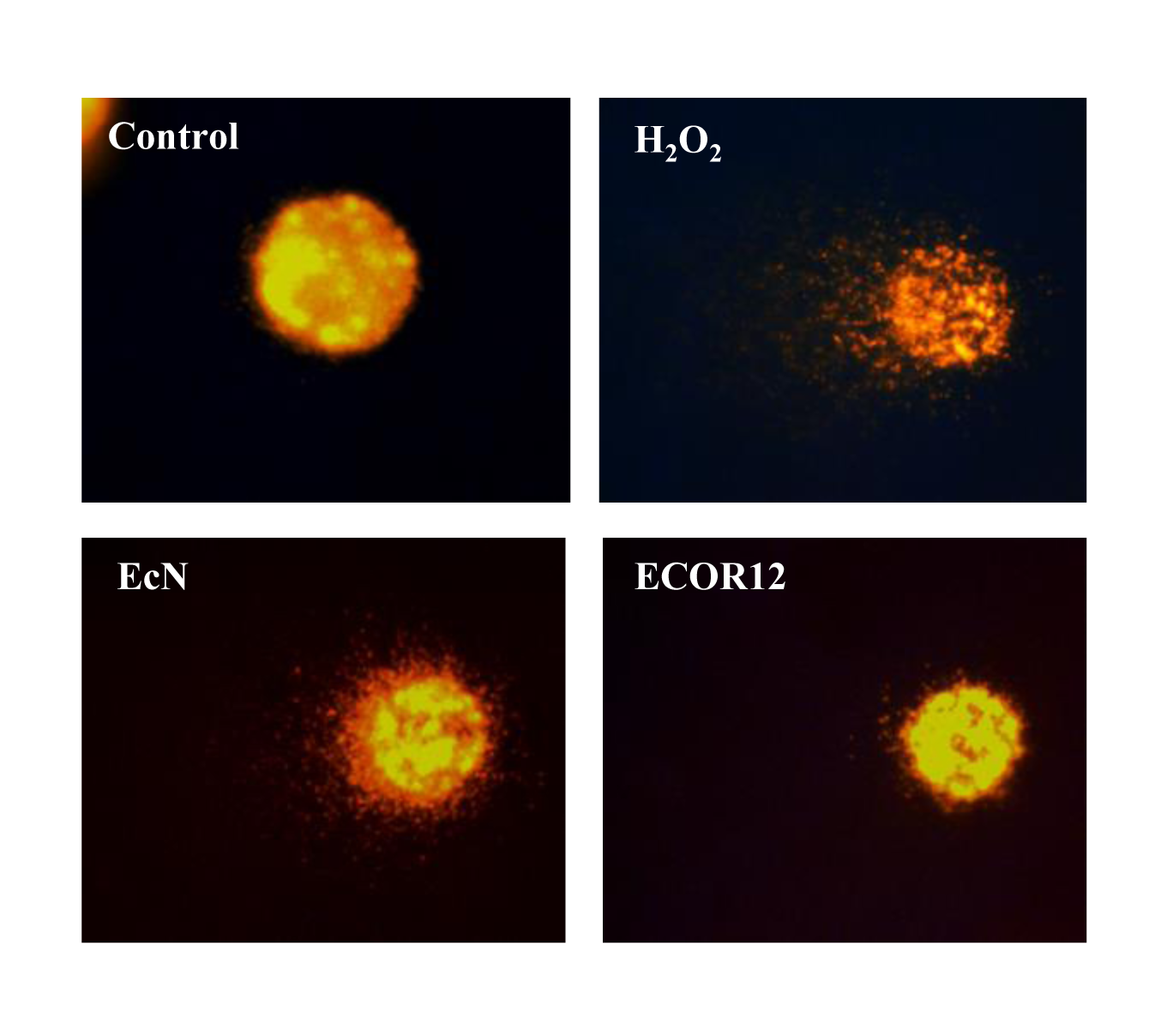

Supplement: S4 Fig — HT-29 cells treated with the indicated OMVs (5 μg/ml) for 48 h or with 300 μM H2O2 for 24 h were trypsinized and processed for alkaline cell-single electrophoresis assay. DNA was stained with ethidium bromide (20 μg/ml). The slides were examined using a Leica D1000 microscope with a 63x oil immersion objective. Images are from a single representative experiment (n = 3). (TIF) [file pone.0160374.s004.tif]
